# Determinants of Under-Five Mortality in an Armed Conflict Setting: Empirical Findings from the Demographic and Health Surveys

**DOI:** 10.3390/ijerph192114179

**Published:** 2022-10-30

**Authors:** Ibraheem M. Karaye, Kahler W. Stone, Jennifer A. Horney

**Affiliations:** 1 Department of Population Health, Hofstra University, Hempstead, NY 11549, USA; 2 Department of Health and Human Performance, Middle Tennessee State University, Murfreesboro, TN 37132, USA; 3 Epidemiology Program, University of Delaware, Newark, DE 19716, USA

**Keywords:** child mortality, insurgency, disaster, Boko Haram, conflict, health, Nigeria

## Abstract

Insurgencies like Boko Haram may impact the physical health and well-being of adults and children living in geographic areas under their political control. However, it is difficult to obtain reliable health data in conflict-affected areas. This study explored the potential to use data from the Demographic and Health Surveys (DHS) to examine the determinants of under-five mortality in Northern Nigeria. Data were derived from DHS conducted before and after the start of the Boko Haram insurgency in 2009. A multi-level mixed effects logistic regression model was used to identify predictors of under-five mortality in an armed conflict setting. Results were reported as adjusted odds ratios (aOR) and 95% confidence intervals (CI). Residence in an armed conflict setting was not significantly associated with under-five mortality (aOR = 1.06; 95% CI: 1.00, 1.10). However, twin gestation (aOR = 3.18; 95% CI:2.96, 3.42), wealth index of family (richest versus poorest: aOR = 0.42; 95% CI: 0.37, 0.47), religion of mother (Islam versus Christianity: aOR = 1.50; 95% CI: 1.43, 1.57); highest educational level of mother (higher versus none: aOR = 0.33; 95% CI: 0.29, 0.37), and parity of mother, significantly predicted death before the fifth birthday. Repeated studies are needed to assess the impact of Boko Haram insurgency on physical health outcomes, particularly in areas where primary data collection is difficult or impossible.

## 1. Introduction

Political instability and extremist insurgencies present significant challenges in collecting vital statistics and conducting regular public health surveillance. For example, the 2018–2019 Kivu Ebola epidemic that is currently occurring in the Democratic Republic of Congo has been difficult to control, in large part due to political violence. Insurgent groups have attacked response personnel, health care providers, and clinics, which interrupted core response activities like identification of Ebola victims, tracing of suspected contacts, and vaccination of at-risk persons. Since the outbreak was declared on 1 August 2018, more than 100 violent attacks have been recorded in the region, including 42 separate attacks on health care facilities and the murder of a World Health Organization (WHO) epidemiologist [1,2]. In Nigeria, Boko Haram has also severely disrupted health services especially in the Northeastern region. Borno—one of the states severely impacted by the insurgency, has lost more than 40% of its healthcare facilities to the insurgency leaving only a third of the remaining facilities fully functional [3]. An estimated 48 health care workers were killed in the state and more than 250 injured. Between 2015 and 2017, 35% of the physician workforce had been lost to other states [3]. Targeting of health facilities has been employed as a tactic of war in Burundi, which severely constrained the supply of health services [4,5] and in Nicaragua between 1982 and 1987, where more than 20% of health units were destroyed [6].

Several studies have addressed the potential for excess morbidity and mortality from diseases during complex emergencies, which frequently include population movement and resettlement, environmental degradation, food shortages, and limited access to healthcare [7,8,9,10,11,12,13]. Insurgencies and other conflicts can intensify these issues by preventing the timely collection of surveillance data and the implementation of control measures [4]. Longer-term political and military conflicts can also be associated with intimate partner violence and limit progress towards the eradication of diseases or sustainable development goals [14,15,16].

Boko Haram, an Islamic sect that seeks to create a “pure” Islamic state (envisaged to be more fair and transparent) in Nigeria, was founded in 2002. In 2009, Boko Haram insurgents increased violent activities in Northern Nigeria after longstanding unrest in the region following the Maitatsine uprisings in Kano State in 1980, and violence in Kaduna and Bulumkutu States in 1982, and Bauchi State in 1985 [17]. In 2010, the scope of attacks in the north-east of Nigeria increased, and included bombings, mass shootings, and executions [17], culminating in the abduction of 276 school girls in 2014 [18]. On 20 January 2012, more than 250 people were killed in bomb attacks in Kano State [19]. According to Human Rights Watch [20], more than 935 people died from terrorism attributed to Boko Haram between 2009 and 2012. 

Insurgencies like Boko Haram affect the health status of residents, particularly children, living in impacted regions in both direct and indirect ways [21,22]. Documented direct effects of the insurgency have included physical [23] and mental health impacts such as Posttraumatic Stress Disorder [24]. Indirect effects include lack of access to both community and community health resources (e.g., adequate shelter, pipe-borne water, food, basic sanitation, and social services); damage to infrastructures like health facilities, roads, markets, water supply, and sewage systems; and disruption of supply chains for food, vaccines, and medication [23,24]. As a result, children exposed to conflicts like this often suffer from higher rates of vaccine-preventable diseases (e.g., measles, polio, hepatitis, and tuberculosis), severe malnutrition, diarrheal disease, and acute respiratory infections. According to the 2013 DHS, the northeastern region of Nigeria has the worst maternal and child health indices in the country [25]. In 2013, more than 50% of the country’s 53 polio cases were from Boko Haram-affected states [26]. In 2014, only a single case of polio was identified in Nigeria, and that was from Borno State, located in the region impacted by Boko Haram [26].

While the acute impacts of Boko Haram attacks on morbidity and mortality can be documented [27], quantifying the longer-term health impacts of this type of political insurgency is difficult. Rapid needs assessments, such as those that can be carried out after a natural disaster or in refugee camps [28] are not possible due to the inability to send teams to the region because of instability on the ground in the regions that are most impacted by Boko Haram activities. Collecting primary data from households to estimate health impacts, as was done after military conflicts in Iraq and Afghanistan [29] is difficult in the region because of sporadic and unpredictable violent attacks (e.g., suicide bombings). Likewise, using data obtained from press accounts may result in inaccurate estimates—first because these data are often incomplete, and second, because these data may not record deaths that are the indirect result of the insurgency. Therefore, in an attempt to address the challenges inherent in collecting data on the health impacts of Boko Haram activities, we used Demographic and Health Surveys (DHS) data to investigate the determinants of under-five mortality in an armed conflict setting in Northern Nigeria. 

## 2. Materials and Methods

### 2.1. Data Sources

Publicly available data were obtained from the 2008 and 2018 Nigeria DHS. Sponsored by the U.S. Agency for International Development (USAID), DHS is a nationally representative household survey that provides data in the areas of population, health, and nutrition [25,30,31]. The survey is conducted every 5 years in more than 90 countries to allow for comparisons over time and across countries. Additional information on DHS is available elsewhere [30,31].

### 2.2. Survey Methodology

A two-stage cluster sampling method was employed by DHS in Nigeria. The country was stratified into six geopolitical zones with urban and rural designations in each zone: North Central, North East, North West, South East, South West, and South South. In each stratum, enumeration areas were determined using data from the most recent national census. In the first stage, primary sampling units were selected from the enumeration areas of each stratum. In the second stage, households within each primary sampling unit were selected. In each selected household, women with children at five or less years old were asked about the health of their children. Following data collection, sampling weights were generated for each primary sampling unit and used in data analysis. A detailed explanation of the survey methodology can be found elsewhere [30,31]. 

### 2.3. Study (Boko Haram-Exposed) and Control (Boko Haram-Non Exposed) States

**Figure 1:**
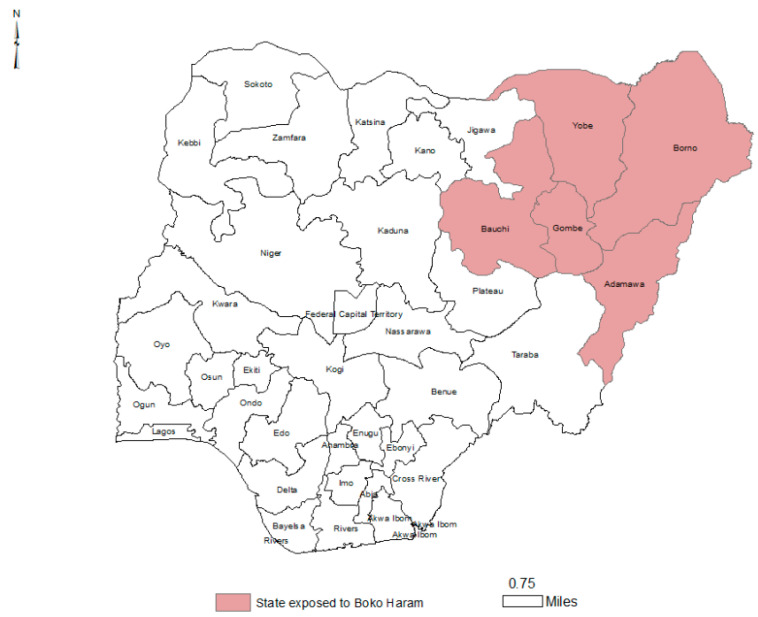
Map of Nigerian States Indicating Boko Haram Presence. (Map generated using ArcGIS 10.7 software, [Esri^©^, Redlands, CA, USA]).

**Figure 2:**
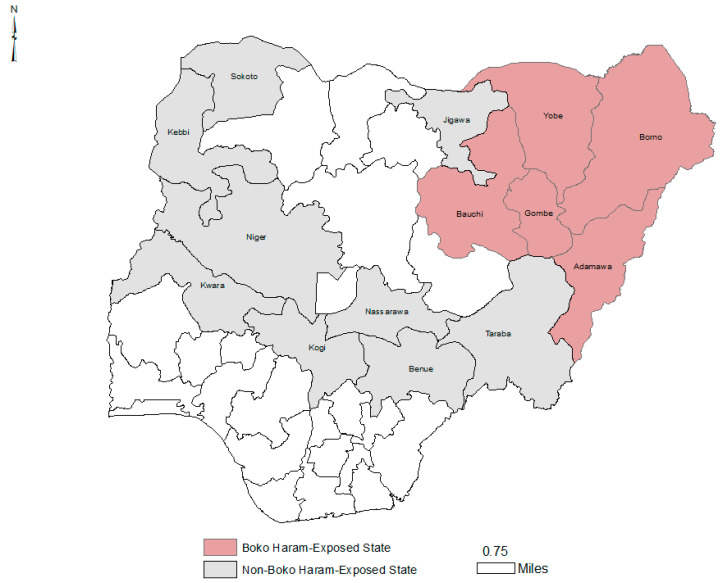
Study and control states. (Map generated using ArcGIS 10.7 software).

For this study, Boko Haram states were defined as those with high Boko Haram activity based on media reports, and included Borno, Yobe, Adamawa, Gombe, and Bauchi (Figure 1). Control states shared similar socio-demographic characteristics, including religion and culture, with the study states except for exposure to the insurgency, and included Sokoto, Jigawa, Niger, Nassarawa, Taraba, Kwara, Benue, Kogi, and Kebbi (Figure 2). Four Northern Nigerian states (Kano, Kaduna, Plateau, and Zamfara) were excluded from the study as they were frequently affected by non-Boko Haram conflicts, like tribal and religious clashes. 

### 2.4. Variables

The outcome variable of interest in this study was ‘child death,’ which was binary (1 = yes, 0 = no). The predictor variables/covariates were: residence in Boko Haram state (1 = yes; 2 = no), sex (1 = male; 2 = female), twin (1 = child is a twin; 2 = child is not a twin), wealth index of family (1 = poorest; 2 = poorer; 3 = middle; 4 = richer; 5 = richest), place of residence (1 = urban; 2 = rural), parity of mother (1 = one childbirth; 2 = two childbirths; 3 = three childbirths; 4 = four childbirths; 5 = five childbirths; six = at least six childbirths), highest educational level of mother (1 = none; 2 = primary; 3 = secondary; 4 = higher), and age of mother (1 = less than 35 years; 2 = at least 35 years).

The variable, wealth index, was derived from data on household ownership of assets, types of water access and sanitation facilities; and materials used for housing construction. Principal component analysis was then used to measure all the households on a continuous scale of wealth, placing them into five quintiles (1 = poorest; 2 = poorer; 3 = middle; 4 = richer; 5 = richest).

### 2.5. Statistical Analysis

DHS data are clustered by design, with individual-level variables (for mother and child) nested within household-level variables. This violates the independence assumption required of an ordinary least squares regression model, as the odds that child death equals one, rather than zero, may vary from one cluster to another, resulting in a Type I error. We fitted two-level mixed effects logistic regression model to account for this potential limitation. A mixed effects logistic regression is suitable for modeling a binary outcome variable in clustered or longitudinal data [32]. The model is parametrized to permit flexibility in the violation of dependence within and between clusters [32]. We used the *xtmelogit* command to model the log odds of child death- as a linear combination of the predictor variables. Sex, twin, age of mother, parity of mother, and highest educational level of mother were included as individual-level categorical predictor variables. Household-level variables comprised the wealth index of family and place of residence. Results were reported as adjusted odds ratios (aOR) and 95% confidence intervals (CI).

All statistical analyses were conducted using Stata 17.0 (College Station, TX, USA). 

## 3. Results

A total of 107,976 births were included in the study, with 49,627 occurring in the five years preceding 2008, before Boko Haram, and 58,349 between 2013 and 2018, after Boko Haram. A larger proportion of respondents in Boko Haram states were educated and wealthy in 2018 as compared to 2008 (higher education: 2.6% versus 1.1%; Richest quintile: 3.8% versus 2.2%) (Table 1). 

Compared to control states, mothers in Boko Haram-impacted states were younger, poorer, less educated, and lived in a rural location. Across both 2008 and 2018 time periods, the proportion of mothers with parity of at least six was higher in Boko Haram states compared to controls, e.g., in 2008, 64.4% of mothers in Boko Haram impacted states had experienced at least 6 childbirths versus 57.2 % of mothers in control states. States exposed to Boko Haram had a higher proportion of child deaths in the period preceding the insurgency than after (24.0% versus 15.8% respectively) (Table 1). 

### Predictors of Under-Five Mortality

Residence in a Boko Haram state was not significantly associated with under-five mortality (OR = 1.06; 95% CI: 1.00, 1.10). Twins were more than three times likely to die than singletons (OR = 3.18; 95% CI: 2.96, 3.42), and the odds of child death increased with the number of children ever born to a mother. For example, a mother that experienced two childbirths was 1.4 times as likely to record under-five child mortality than a mother who had given birth once (OR = 1.44; 95% CI: 1.24, 1.67). Likewise, a mother that has given birth at least six times was nearly three times as likely to record under-five mortality than one who had only conceived once (OR = 2.94; 95% CI: 2.57, 3.36). Wealth was found to be protective, as children raised in a wealthier family were less likely to die before their fifth birthday than the socioeconomically deprived (Richest versus Poorest: OR = 0.42; 95% CI: 0.37, 0.47) (Table 2).

## 4. Discussion

This study used DHS data to examine the determinants of under-five mortality in an armed conflict setting in Northern Nigeria. The study used available secondary data because reliable primary data collection is difficult in conflict-affected areas, where health data are badly needed due to the potential for conflict to have both direct and indirect impacts on physical and mental health of residents. Using the available data, residence in an armed conflict setting was not significantly associated with under-five mortality. Twin gestation, wealth index of family, study period, age of mother, highest educational status of the mother, sex of the child, mother’s religion, and parity of mother predicted deaths before the fifth birthday. 

This study did not find a significant association between residence in an armed conflict setting and under-five mortality. There are potential explanations for this observation. During the sampling phase of the DHS survey, some clusters considered high risk of insurgent activity were excluded due to safety concerns [25,33]. This exclusion might have selectively biased the results seen in this study. For example, if respondents more likely to need access to health care impacted by Boko Haram activities were less likely to be included, this could bias estimates towards the null. For example, the share of the ‘richer’ quintile in the Boko Haram region was 10.2% in 2018 as opposed to 8.2% in 2008. In 2018, the ‘richest’ quintile in the Boko Haram region was 3.8% as opposed to 2.8% in 2008, which might indicate selection bias (Table 1). Second, DHS only interviewed alive mothers at the time of the survey [31]. Thus, deceased mothers of deceased children were excluded from the study, which might also bias estimates towards the null. Third, Boko Haram predominantly targeted the state, its institutions (e.g., police stations, army barracks), and public places (e.g., markets and places of worship)—locations with higher adult populations than children under five years of age [33]. Consequently, deaths resulting from direct exposure to the insurgent attacks would be expected to be lower in under-fives than in older age groups. Fourth, given that children under-five have better innate immunity and higher immunologic memory to survive recurrent infections compared to younger children (like infants and neonates) [34], they would be expected to better tolerate the indirect health impact of the insurgency (like vaccine preventable diseases, diarrheal disease, acute respiratory infections, and severe malnutrition), which might otherwise increase their likelihood of mortality. Fifth, the rising positive social change occurring in Northern Nigeria, such as increasing age at marriage and higher educational attainment, could help reduce under-five mortality even in an insurgency era [35,36].

The positive association between twin births and under-five mortality may be explained by the higher incidence of pre-term birth, intrauterine growth restriction, twin-twin transfusion syndrome, and congenital abnormalities seen in twin gestation and therefore a higher rate of morbidity and mortality [37,38]. As much as a 3-fold increase in the perinatal risk of death has been estimated to occur in a twin, compared to a singleton pregnancy [39]. The finding that wealth index was negatively associated with under-five mortality is consistent with a previous study, which found that 16% of children in poor households die before their fifth birthday, compared to 8% of children born in wealthy households [40]. The influence of wealth on child morbidity and mortality may be explained by the effect of income on proximate determinants of health- food, water, clothing/bedding, housing, fuel/energy, transportation, hygiene/preventive care, sickness care, and information [41].

The positive association between parity and child mortality is well documented in the literature [42,43]. Various theories have been proposed to explain this observation, ranging from biologic factors affecting the mother to sociologic factors that affect access to care and utilization of health care services [44]. The biologic theory posits that increased physical and caloric stress associated with high parity results in a ‘maternal depletion syndrome’, which subsequently affects the health and wellbeing of the child [44]. The sociologic theory postulates that children of high parity mothers suffer from poor nutritional status, and subsequently higher mortality, due to reduced parental investment and competition between siblings for finite resources [45].

This study has several limitations. As previously discussed, eight clusters in areas designated as exposed to Boko Haram were deliberately omitted from the DHS survey in 2013 due to security concerns [25], which might have selectively biased the study results. Future studies should be conducted using primary data, where it can be safely collected to assess this research question. Other approaches to collecting valid public health data in areas impacted by violence should also be considered. Second, the migration history of families was not captured in the data. It is possible that families with higher child mortality might have migrated to other neighboring countries (e.g., Chad) or the Nigerian States that were perceived to be relatively safer and were therefore not included in this study. Third, historical data on childbirth and death were obtained by DHS using self-reported questionnaires, which might introduce recall bias, although the death of a child before age 5 is unlikely to be misremembered by a survey respondent. However, misreporting of child death is still possible- mothers may not wish to discuss the death of a child and thus decide not to mention it during a survey. 

Notwithstanding these limitations, our study has considerable strengths. Our findings are an important contribution to the disaster literature, which is grossly deficient in the determinants of under-five mortality in armed conflict settings. The predictors identified by our study could be used to direct evidence-based intervention efforts for improving child survival in disaster settings. Second, we employed a multilevel mixed effects logistic regression that is suitable for clustered DHS data. The use of ordinary least squares regression would have disregarded the independence assumption, resulting in a Type 1 error, and limiting the validity of our results. Finally, the data for this study were derived from DHS, which has been proven to be an accurate and reliable source of nationally representative data for developing countries [46].

## 5. Conclusions

Residents of armed conflict settings are not uniformly impacted by under-five mortality. In the face of limited public health resources, culturally attentive interventions should be directed at twin children, poor households, multiparous women, and mothers aged over 35 years, with little or no western education. In addition, further studies should consider collecting primary or rapid assessment data from Boko Haram-afflicted areas to re-examine the impact of the insurgency on physical health outcomes.

## Figures and Tables

**Table 1:** Descriptive Statistics of Select Variables.

	Before Boko Haram (2008)		After Boko Haram (2018)	
	Boko Haram-Exposed State *n* = 20,099 (%)	Control State *n* = 29,528 (%)	Boko Haram-Exposed State *n* = 22,402 (%)	Control State *n* = 35,947 (%)
Sex				
Male	10,377 (51.6)	15,231 (51.6)	11,648 (52.0)	18,259 (50.8)
Female	9722 (48.4)	14,297 (48.4)	10,754 (48.0)	17,688 (49.2)
Parity of Mother				
One	542 (2.7)	915 (3.1)	647 (2.9)	1071 (3.0)
Two	1090 (5.4)	1938 (6.6)	1336 (6.0)	2242 (6.2)
Three	1497 (7.5)	2823 (9.6)	1908 (8.5)	3249 (9.0)
Four	1952 (9.7)	3208 (10.9)	2304 (10.3)	3984 (11.1)
Five	2075 (10.3)	3770 (12.8)	2590 (11.6)	4470 (12.4)
At least Six	12,943 (64.4)	16,874 (57.2)	13,617 (60.8)	20,931 (58.2)
Highest Educational Level of Mother				
No education	15,931 (79.3)	20,984 (71.1)	16,654 (74.3)	23,748 (66.1)
Primary	2768 (13.8)	5285 (17.9)	2879 (12.9)	5961 (16.6)
Secondary	1173 (5.8)	2498 (8.5)	2288 (10.2)	4856 (13.5)
Higher	227 (1.1)	761 (2.6)	581 (2.6)	1382 (3.8)
Religion				
Islam	17,363 (86.4)	19,539 (66.2)	19,493 (87.0)	26,863 (74.7)
Christianity/Traditional	2588 (12.9)	9675 (32.8)	2905 (13.0)	9081 (25.3)
Missing	148 (0.1)	314 (1.1)	4 (0.0)	3 (0.0)
Child is Twin				
Yes	554 (2.8)	1015 (3.4)	556 (2.5)	1209 (3.4)
No	19,545 (97.2)	28,513 (96.6)	21,846 (97.5)	34,738 (96.6)
Place of Residence				
Rural	15,258 (75.9)	24,452 (82.8)	17,395 (77.7)	28,272 (78.7)
Urban	4841 (24.1)	5076 (17.2)	5007 (22.4)	7675 (21.4)
Death of Child				
Yes	4829 (24.0)	5957 (20.2)	3549 (15.8)	6208 (17.3)
No	15,270 (76.0)	23,571 (79.8)	18,853 (84.2)	29,739 (82.7)
Wealth Index				
Poorest	9728 (48.4)	9848 (33.4)	9913 (44.3)	11,277 (31.4)
Poorer	5280 (26.3)	8133 (27.5)	5681 (25.4)	9940 (27.7)
Middle	3009 (15.0)	6131 (20.8)	3677 (16.4)	7613 (21.2)
Richer	1644 (8.2)	3617 (12.3)	2292 (10.2)	4868 (13.5)
Richest	438 (2.2)	1799 (6.1)	839 (3.8)	2249 (6.3)
Age of Mother				
Less than 35 years	9264 (46.1)	12,696 (43.0)	10,136 (45.3)	15,177 (42.2)
At least 35 years	10,835 (53.9)	16,832 (57.0)	12,266 (54.8)	20,770 (57.8)

**Table 2:** Crude and Adjusted Estimates of Predictor Variables on Child Mortality.

	Crude Model OR (95% CI)	Adjusted Model OR (95% CI)
Residence in Boko Haram-Exposed State (Ref = “No”)		
Yes	* 1.07 (1.04, 1.11)	1.06 (1.00, 1.10)
Sex (Ref = “Male”)		
Female	* 0.93 (0.90, 0.96)	* 0.93 (0.90, 0.96)
Child is a Twin (Ref = “No”)		
Yes	* 3.33 (3.11, 3.58)	* 3.18 (2.96, 3.42)
Wealth Index (Ref = “Poorest”)		
Poorer	* 0.91 (0.88, 0.95)	* 0.93 (0.90, 0.97)
Middle	* 0.68 (0.65, 0.71)	* 0.74 (0.70, 0.77)
Richer	* 0.51 (0.48, 0.54)	* 0.57 (0.53, 0.61)
Richest	* 0.33 (0.30, 0.37)	* 0.42 (0.37, 0.47)
Parity of Mother (Ref = “One”)		
Two	* 1.46 (1.26, 1.69)	* 1.44 (1.24, 1.67)
Three	* 1.47 (1.27, 1.69)	* 1.44 (1.25, 1.66)
Four	* 1.68 (1.46, 1.93)	* 1.60 (1.39, 1.84)
Five	* 1.94 (1.69, 2.22)	* 1.81 (1.58, 2.08)
At least Six	* 3.50 (3.07, 3.99)	* 2.94 (2.57, 3.36)
Age of Mother (Ref = “<35 years”)		
At least 35 years	* 1.46 (1.42, 1.51)	* 1.05 (1.01, 1.09)
Highest Educational Level of Mother (Ref = “None”)		
Primary	* 0.78 (0.75, 0.82)	0.98 (0.94, 1.03)
Secondary	* 0.47 (0.44, 0.50)	* 0.91 (0.85, 0.98)
Higher	* 0.33 (0.29, 0.37)	* 0.80 (0.69, 0.92)
Religion (Ref = “Christianity/Traditional”)		
Islam	* 1.61 (1.54, 1.67)	* 1.50 (1.43, 1.57)
Place of Residence (Ref = “Urban”)		
Rural	* 1.43 (1.37, 1.48)	1.05 (1.00, 1.10)

* *p*-value < 0.05.

## Data Availability

Data for this study can be accessed from https://dhsprogram.com/data/ (accessed on 26 October 2022).
